# Anodization-based process for the fabrication of all niobium nitride Josephson junction structures

**DOI:** 10.3762/bjnano.8.58

**Published:** 2017-03-02

**Authors:** Massimiliano Lucci, Ivano Ottaviani, Matteo Cirillo, Fabio De Matteis, Roberto Francini, Vittorio Merlo, Ivan Davoli

**Affiliations:** 1 Dipartimento di Fisica and MINAS Lab, Università di Roma Tor Vergata, 00133 Roma, Italy; 2 CNR-SPIN Institute, Via Giovanni Paolo II, 84084 Fisciano (SA), Italy; 3 Dipartimento di Ingegneria Industriale, Università di Roma “Tor Vergata”, Via del Politecnico, 00133 Roma, Italy

**Keywords:** Josephson effect, superconductors, thin films, tunneling

## Abstract

We studied the growth and oxidation of niobium nitride (NbN) films that we used to fabricate superconductive tunnel junctions. The thin films were deposited by dc reactive magnetron sputtering using a mixture of argon and nitrogen. The process parameters were optimized by monitoring the plasma with an optical spectroscopy technique. This technique allowed us to obtain NbN as well as good quality AlN films and both were used to obtain NbN/AlN/NbN trilayers. Lift-off lithography and selective anodization of the NbN films were used, respectively, to define the main trilayer geometry and/or to separate electrically, different areas of the trilayers. The anodized films were characterized by using Auger spectroscopy to analyze compounds formed on the surface and by means of a nano-indenter in order to investigate its mechanical and adhesion properties. The transport properties of NbN/AlN/NbN Josephson junctions obtained as a result of the above described fabrication process were measured in liquid helium at 4.2 K.

## Introduction

Niobium (Nb) is the most commonly used material in superconducting electronics [1–3], but several groups have been investigating the properties of metals and alloys that could represent an alternative to it. Niobium nitride (NbN), in particular, is a promising material in this respect given its relatively high critical temperature and energy gap of the order, respectively, of 16 K and 2.5 mV [4–8]. The transition temperature is appealing because of the progress achieved in closed-cycle refrigeration, while the value of the gap is stimulating for engineering devices in the terahertz range. Several papers have been dedicated in the past to the realization of all-NbN-based superconducting tunnel junctions [9–11], but a reliable technology generating samples with quality features similar to the ones of all-Nb samples [1,12] has not emerged yet.

We have undertaken a systematic study of the production of all-NbN films and all-NbN tunnel junctions. In a previous publication the authors of the present paper investigated the effect of the substrate on the quality of the NbN films and the NbN/AlN interface [13] while preliminary lithographic attempts on NbN/AlN/NbN trilayers were presented in another publication [14]. In the present paper our effort toward the realization of high-quality all-NbN tunnel junctions continues. We have first set up the conditions for a highly reproducible and controllable growth of good-quality NbN monitoring the sputtering plasma by optical spectroscopy [15–17]. The same technique has been employed to obtain AlN thin films. At first, we have decided to concentrate on AlN as dielectric layer in NbN/AlN/NbN junctions because this material seems to be the most promising at present. Moreover, as a first step we considered reasonable to match our overall fabrication recipe with existing processes and literature. Along with the optimal and controlled film growth we also carried out an investigation of the anodization of these films since this technique is necessary for the most of the procedures leading to the patterning of tunnel junctions.

The processes for defining patterns on NbN thin films are typically based on ion etching and subsequent deposition of insulating layers [9–11]. We have tried to limit our fabrication recipe instead to minimal procedures, namely just lift-off lithography and selective anodization. By using lift-off lithography aggressive and high-energy etching processes such as ion milling and reactive ion etching (RIE) can be avoided. The use of anodization can reduce the number of mask and photolithography steps. In particular, it is not necessary to deposit further insulators to separate different metals in multilayers. In all of our investigations the substrates are commercially available silicon wafers coated with 1 μm artificial oxide. This choice was also dictated by the perspective of setting up a recipe that could well lend itself to be developed for “large-scale” commercial applications.

## Experimental

### Methods

One reliable process to deposit NbN and AlN films consists of using a reactive sputtering chamber [4–8]. When such a technique is employed, however, the quality of the films depends on several parameters: the purity of the deposition chamber, the reciprocal positions of source and substrate, the relative pressure of N_2_, the magnetic field, the dc or the rf power used and the surface temperature of the substrate. The last condition is particularly critical because the increase of the kinetic energy of ions close to the substrate promotes the formation of nitrides, but deteriorates the vacuum conditions. Temperature control is particularly relevant when lithographic patterns impressed by photoresist (an organic polymeric compound melting above 120 °C) are present on the substrate because the degassing of the polymer pollutes the deposition chamber. For this reason the temperature of the substrate must be kept constantly below 120 °C. This often makes it necessary to alternate periods of sputtering with periods in which the sputtering is stopped.

Eye inspection of the plasma during the sputtering reveals that the color of the glow discharge changes from blue to red with increasing nitrogen concentration. A quantitative analysis of this effect can be obtained by relating the concentration of N_2_ in the plasma to the intensities of the emission lines observed with the aid of a spectrometer [15–17]. This technique has enabled us to link the plasma composition and the final composition of the nitride films. To our knowledge, this is the first time this optical spectrometry technique is applied in the production of superconductive metallic films for optimizing the transition temperature and film quality. Since the deviation from a stoichiometric composition of the NbN affects (reducing it) the superconducting critical temperature, the pattern of the emission lines in the glow discharge provides direct information on the critical temperature of the deposited nitride films. For AlN the gas composition affects the resistivity of the film and, even in this case, the pattern of the emission lines in the glow discharge provides information on the final resistivity of the sputtered films.

The anodization of the NbN layer was obtained by means of a 1 mol solution of ammonium pentaborate in ethylene glycol. In order to oxidize NbN samples, we used a current-control technique, i.e., the current density has been set while the potential difference (*V*_s_) between cathode and anode in the electrolytic cell has been monitored simultaneously. The samples were anodized using different current bias and voltage compliance (*V*_c_) to scrutinize the effects on the growth of NbN oxide. The cell has a platinum electrode at fixed distance of 1 cm parallel to the sample surface.

A systematic study on different thicknesses (depending on *V*_s_) was performed to obtain a compact layer without defects and cracks. The anodized films were also characterized by Auger spectroscopy to analyze compounds formed on the surface and their stoichiometry. A multilayer superconductor/insulator/superconductor was obtained by successive depositions and patterned by means of the proposed anodization technique to obtain a NbN/AlN/NbN Josephson junction the current–voltage characteristic of which was measured in a liquid helium bath at 4.2 K.

### Equipment

The sputtering system used in our experiment is a commercial Leybold sputtering system equipped with two 4 inch dc and rf magnetron sources and one etching source. A flux meter controls the inlet for N_2_ and Ar. Finally, a rotatable disk plate holds several substrates in the chamber; this plate is grounded to allow for ion cleaning before the sputtering process. For the deposition of NbN, the dc mode was used with different gas mixtures of N_2_/Ar flux, (total pressure 5 × 10^−1^ Pa), while AlN was deposited in rf mode (150 W) with a gas mixture of N_2_ and 20 sccm of Ar, the base pressure in the chamber being 10^−4^ Pa. In these conditions, the deposition rates for NbN and AlN were 0.6 nm/s and 0.06 nm/s, respectively.

The calibration of the deposition rate was performed by measuring, ex situ, the thickness of the obtained film by means of a profilometer. All the samples described in this work were grown on Si(111) substrates covered by 1 μm of amorphous, artificially grown oxide. Following common recipes reported in literature the target–substrate distance is about 10 cm and the substrate temperature, measured by a thermocouple, is kept below 50 °C during the NbN film growth. Alternating flash deposition of 120 s with pauses of about 20 s was carried out. The thickness of the deposited films was 300 nm.

The critical temperature *T*_c_ of the NbN thin films has been determined by four-probe measurements of resistance as a function of the temperature with the samples secured to the cold finger of a Gifford–McMahon cryocooler. The characteristics of the films were measured as a function of two parameters of the deposition process: the dc power of the source (increasing or reducing the rate of the flash and pause during sputtering) and the concentration of N_2_ in the gas mixture present during the sputtering process. Under these conditions, we obtained films with different superconducting transition temperatures (*T*_c_) ranging from 9.0 to 15.5 K. The room-temperature sheet resistance (resistivity per unit thickness) of the AlN films was also measured by using the four-probe technique. Different AlN specimens have been prepared, keeping constant the rf power (150 W) and the argon flux (20 sccm), but varying the nitrogen concentration during the sputtering process.

The optical emission spectra of the plasma discharge were acquired with an Ocean Optics Spectrometer, model HR 4000, in the range of 300–1000 nm, connected to the vacuum chamber by means of an optical fibre pointing directly into the plasma. The measurements of hardness, reduced modulus and surface roughness have been performed by Nano Test Micro Materials Ltd., using a diamond Berkovich tip.

## Results and Discussion

### NbN films

**Figure 1:**
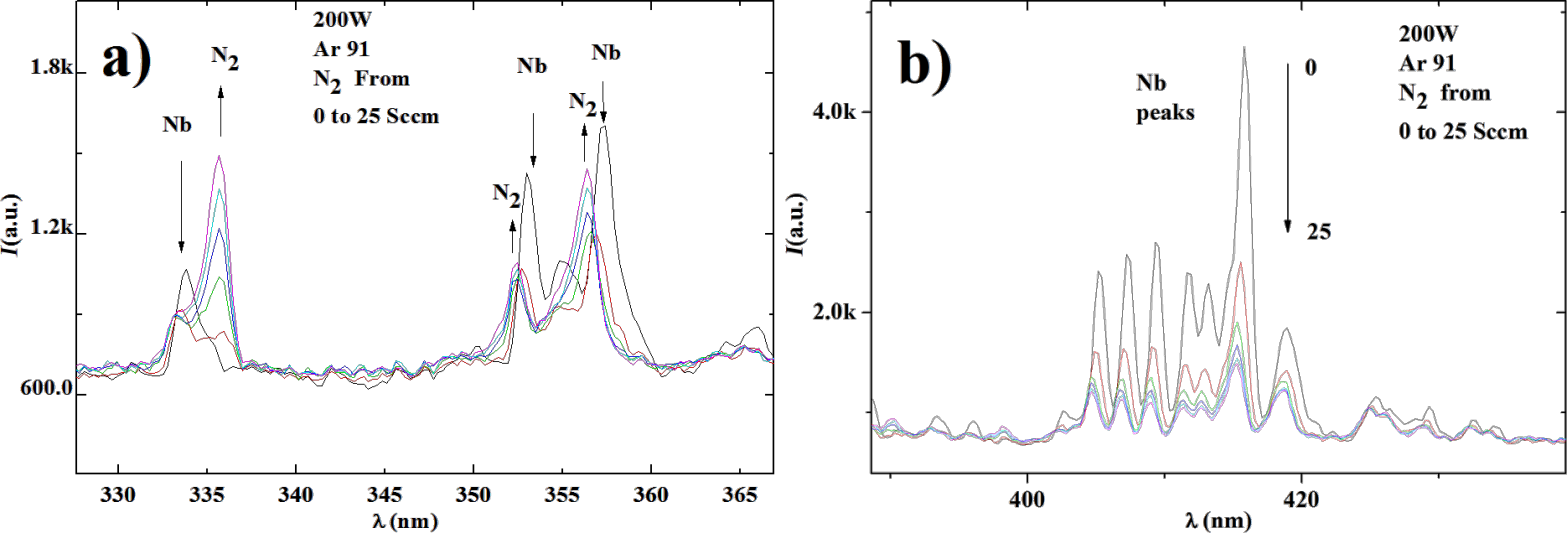
a) Glow-discharge peak intensity monitored in the region of 328–367 nm, an energy region in which we find both Nb and N_2_ peaks. In the inset the power, the fixed Ar concentration and the range of the amount of N_2_ are reported. The color of the six curves, obtained for increasing values of the N_2_ flux, starting from 0 and going up to 25 sccm in steps of 5 sccm are as follows: black, red, green, dark blue, cyan, and magenta; b) Glow-discharge peak intensity in the region 388–438 nm, inset as in panel a.

In Figure 1 we show two optical emission spectra of the glow discharge in a selected wavelength range as detected during the preparation of the NbN deposition, just before opening the shutter. Both curves show the intensity of the light emission peaks as a function of the wavelength, detected during the glow discharge for a fixed power of 200 W. The data show the trend of the intensity of the N_2_ and of Nb peaks, when the gas content of the chamber changes from a constant flux of Ar (91 sccm) to a mixture of N_2_/Ar obtained by increasing the N_2_ injection from 0 to 25 sccm in steps of 5 sccm. We note that the intensity of the N_2_ peak (see the peak at 337 nm) increases to a value 2.5 times that of the background (600 a.u.), while the Nb peak (416 nm) decreases to about half of its maximum value. This happens because niobium reacts with N_2_ to the nitride. The more N_2_ is present the higher is the amount of nitride generated and the lower is the amount of Nb atoms emitting at a given spectral position. The relative height of the Nb and N_2_ peaks is the parameter that we correlate to the transition temperatures (and quality) of our NbN films. In order to do so we always select the spectral lines exhibiting the largest variation as a function of the nitrogen inlet. In this specific case (Figure 1), we select the lines mentioned before, namely 337 nm for N_2_ and 416 nm for Nb.

**Figure 2:**
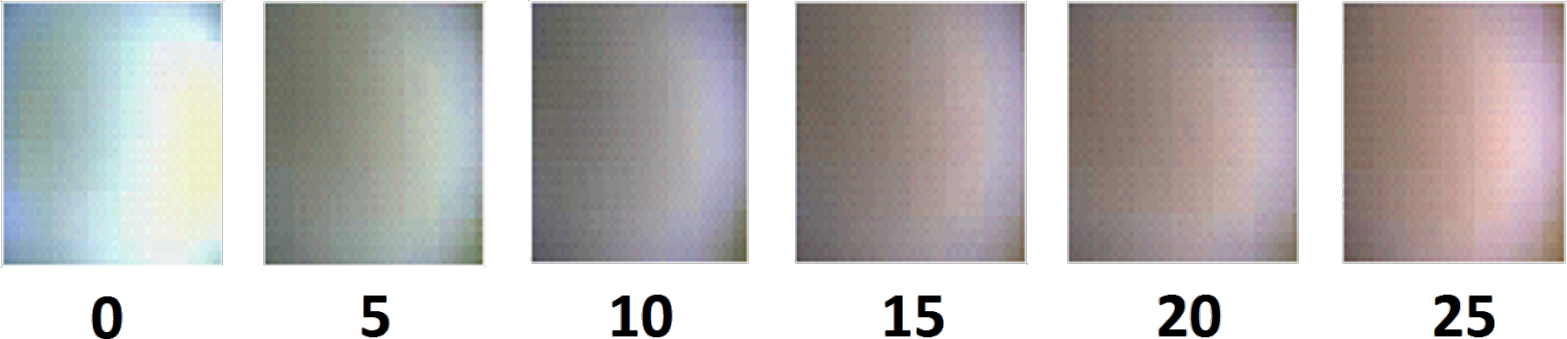
The color of the plasma corresponding to the six increasing values of the input flux described in Figure 1 (0 sccm is the leftmost, 25 sccm is the rightmost).

Figure 1 also shows how to quantify the color change visible by eye in the plasma and obtain a fine tuning of the composition of the plasma during the sputtering deposition. Each of the curves shown in Figure 1 corresponds to a different concentration of N_2_ and to different colors, which are shown in Figure 2.

**Figure 3:**
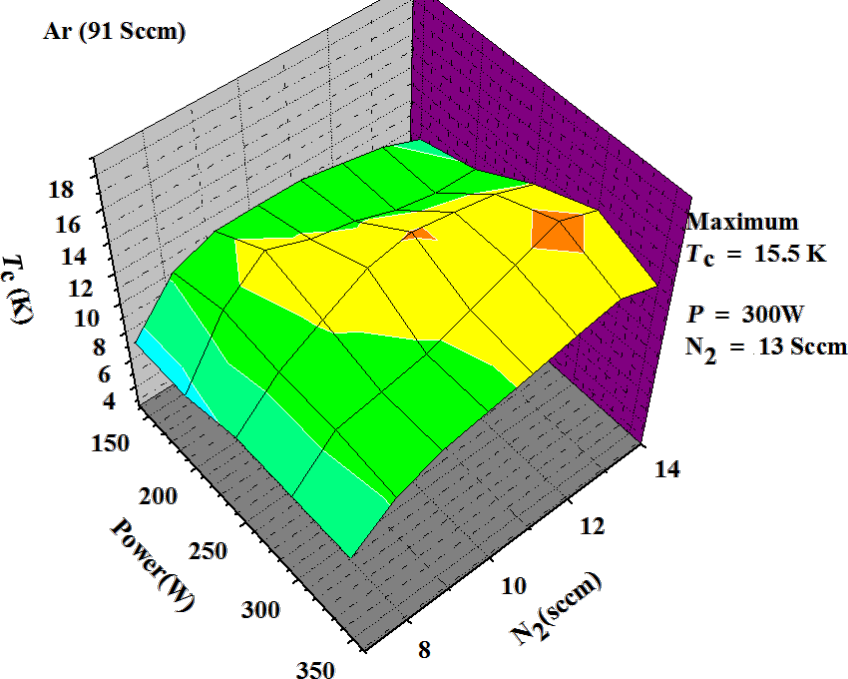
3D plot constructed to optimize sputter deposition. Each point of the grid corresponds to a sample fabricated with the given set of parameters. We can see that in our apparatus with a basic pressure of 2 × 10^−5^ Pa the highest *T*_c_ (15.5 K) is obtained with 300 W and a gas mixture of 14% N_2_ in Ar.

In Figure 3 we report a three-dimensional plot showing the values of critical temperature (*T*_c_) of NbN films as a function of the dc power applied on the target and the N_2_ flux inlet in the deposition chamber for 40 different recipes. *T*_c_ is the temperature for which the electrical resistance, measured with the four-point probe technique, drops to zero. We have fabricated more than 100 films with a thickness maintained constant at about 300 nm. Several points in the plot of Figure 3 were repeatedly measured with a variance of the values of the critical temperature within few tenths of a degree.

**Figure 4:**
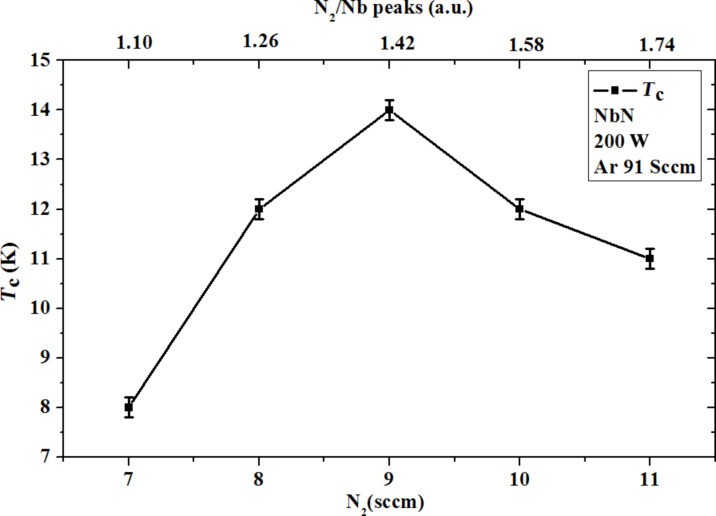
Critical temperature *T*_c_ of NbN as a function of the N_2_ flux and of the of N_2_/Nb peak ratio. In the inset the deposition parameters are given.

For each film, we have varied either the dc power or the concentration of N_2_ in the chamber. We note that the film with the highest critical temperature (15.5 K) is obtained when the N_2_ flux is 13 sccm and the dc power applied is 300 W. Unfortunately, high power on the target implies a high temperature on the substrate. Consequently, in order to deposit NbN films on a patterned mask, we must use a lower dc power, and find another concentration of N_2_. Because the high temperature induced on the substrate softens the photoresist and pollutes the NbN films. In Figure 4 the section of the 3D-plot of Figure 3 corresponding to a dc power of 200 W is shown. We can see more clearly that a N_2_ flux of 9 sccm gives NbN film with a *T*_c_ of 14 K. In addition from the top axis of Figure 4 we can trace the dependence of *T*_c_ upon the ratio between the height of the peaks on Nb and N_2_. Thus, by monitoring the relative height of the spectrometric peaks, as mentioned above, we can follow the growth of NbN and estimate the expected transition temperature.

Equation 1 gives the fit of the measured *T*_c_ (the surface of Figure 3) as a function of the power (*P*) in watts and the nitrogen flux (*F*) in sccm. We observe a strong dependence of *T*_c_ on the nitrogen flux, a tendency opposite to the data obtained by Dawson-Elli et al. [5] who worked in a saturated regime of nitrogen flux.

[1]
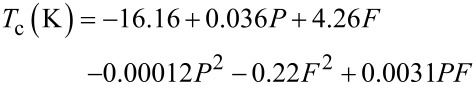


### AlN films

**Figure 5:**
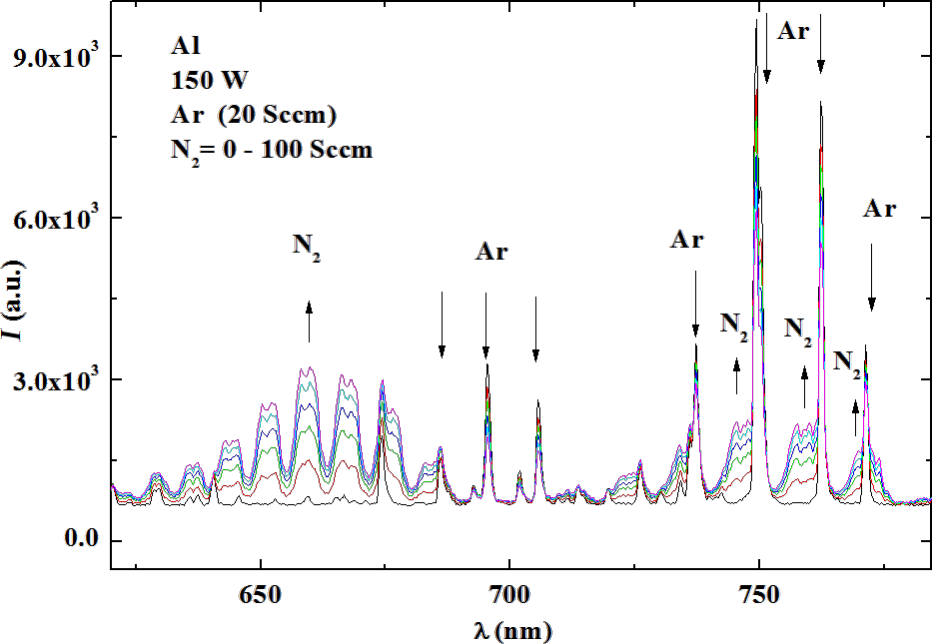
Spectrum of the emission lines. Details of AlN for different values of N_2_ flux (starting from 0 and going up to 100 sccm in steps of 20 sccm, the colors are as follows: black, red, green, dark blue, cyan, and magenta) at fixed power (150 W) and an Ar flux of 20 sccm.

In Figure 5 the relative intensities of the optical plasma emission obtained during the deposition of AlN films, are plotted in the wavelength range of 590–820 nm. The spectra are recorded for a constant rf power of 150 W, while the N_2_/Ar mixture is varied by changing the N_2_ flux from 0 to 100 sccm while keeping the Ar flux constant at 20 sccm. With increasing N_2_ flux the N_2_ peaks increase and the Ar peaks decrease. Similarly to what we did for NbN (Figure 1), we correlated the relative height of two peaks (for N_2_ and Ar) to the conductivity of the film.

**Figure 6:**
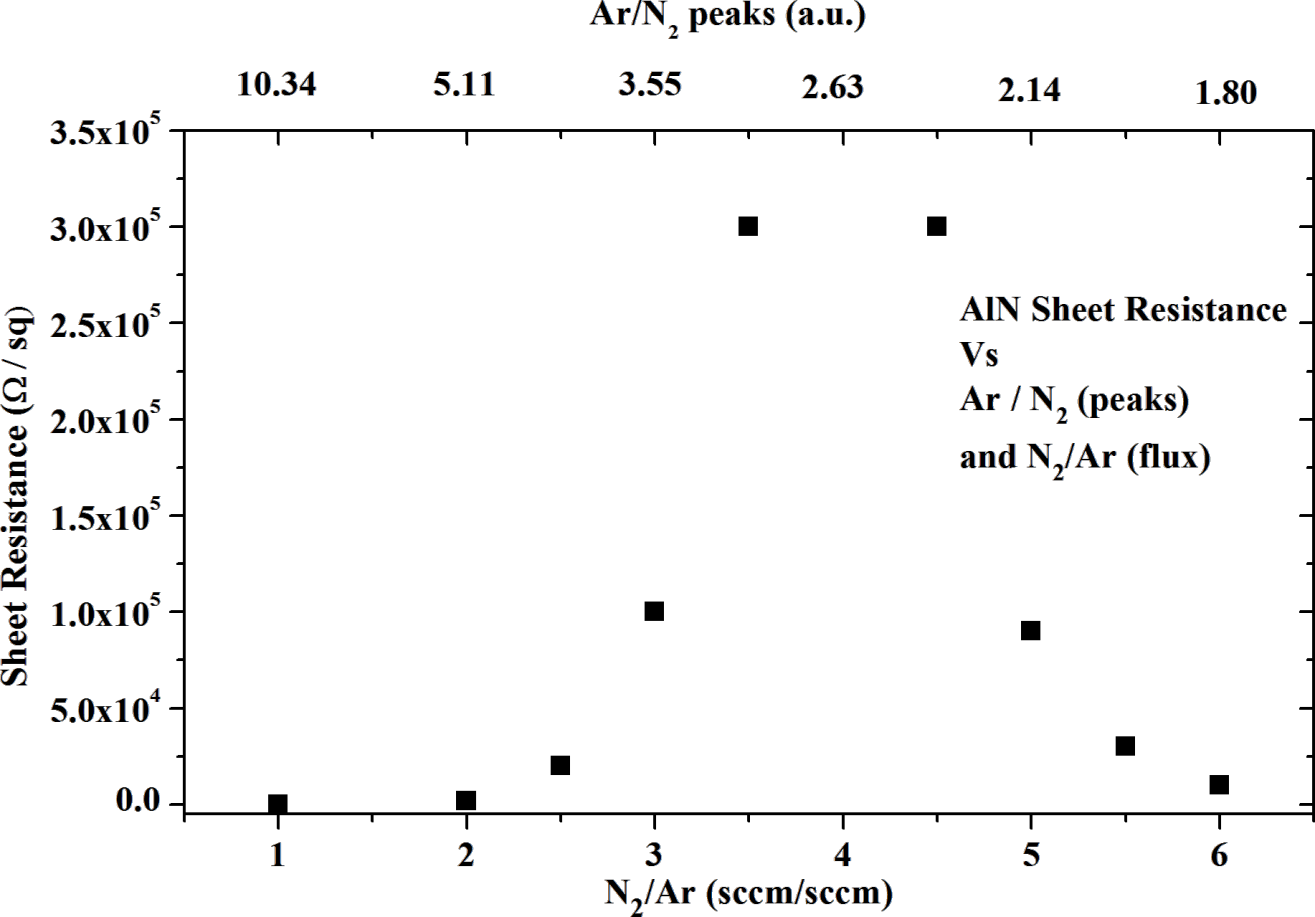
Resistivity of AlN film as a function of N_2_/Ar gas flux and Ar/N_2_ peak ratio. The rf power was fixed at 150 W.

In Figure 6 we plot the sheet resistance of the deposited AlN films as a function of the composition of the N_2_/Ar mixture and of the N_2_/Ar peak ratio. We choose from Figure 5 the peaks with high intensity that were also very responsive to the gas variations of the gas flux (Ar and N_2_ peaks at 750 nm and 660 nm, respectively). The spectral lines of Ar and N_2_ should be close enough to be simultaneously visualized on the spectrometer display. It is impossible to measure the sheet resistance of a highly insulating film. However, we estimated that when the peak ratio of N_2_/Ar is close to 2.63 the resistance is higher than 4.53 × 10^6^ Ω/sq. (10 MΩ is the maximum value that can be measured by our data-acquisition set-up.)

### NbN anodization

**Figure 7:**
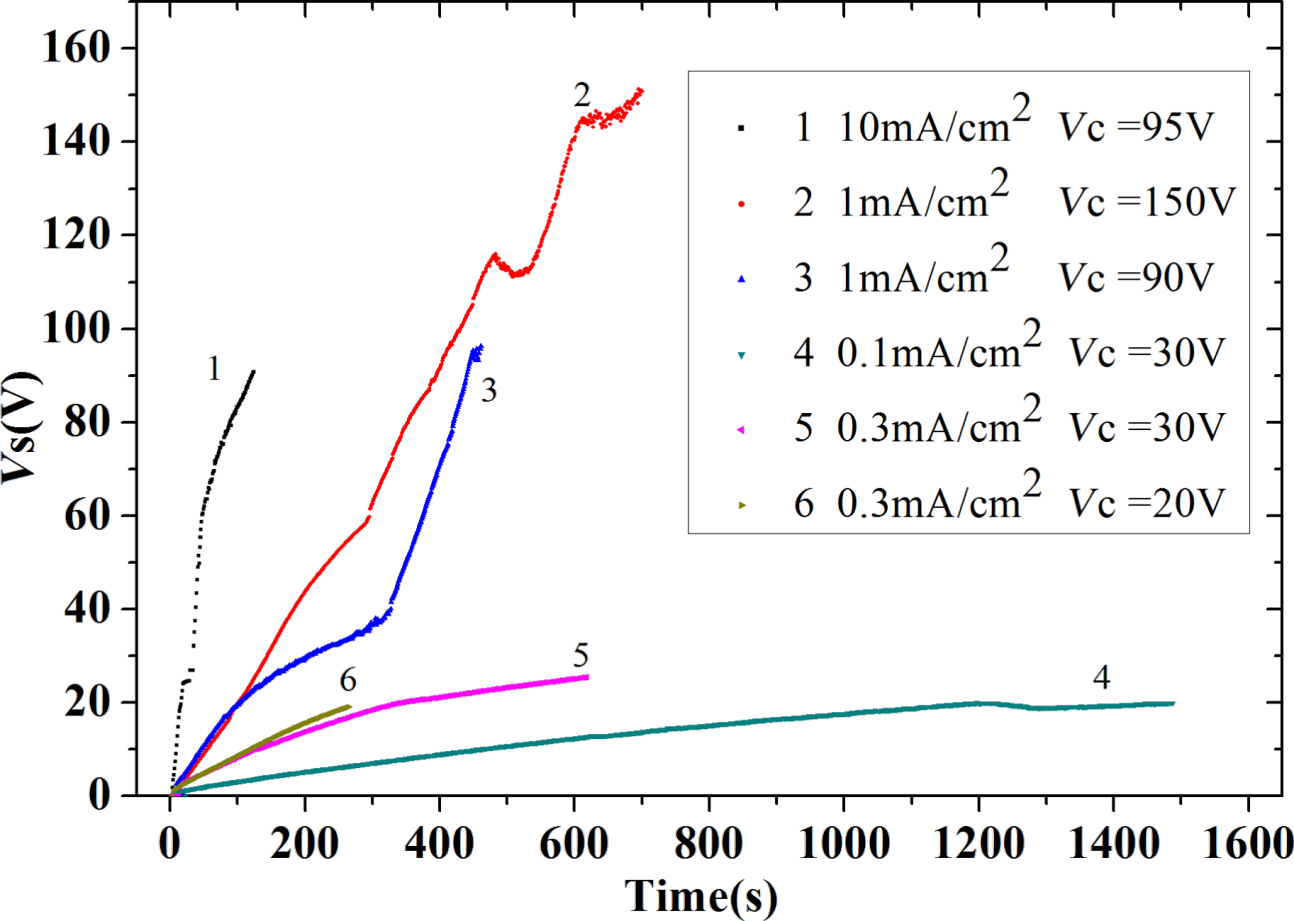
Film voltages as a function of the time obtained varying the current density and compliance voltages for different samples in our NbN anodization process. The number close to each curve is associated with the current and voltage parameters indicated in the inset.

For the anodization recipe we started selecting the parameters generally used to anodize niobium, namely current densities of {10; 1; 0.3; 0.1} mA/cm^2^ at different compliance voltages. In Figure 7 we show the voltage difference between the NbN films and the counter electrode during the anodization process as a function of time. The inset indicates the anodization currents and voltages. We observed that a voltage noise is present at high current densities (curves 1,2,3) that reduces *V*_c_ from 150 to 90 V.

**Figure 8:**
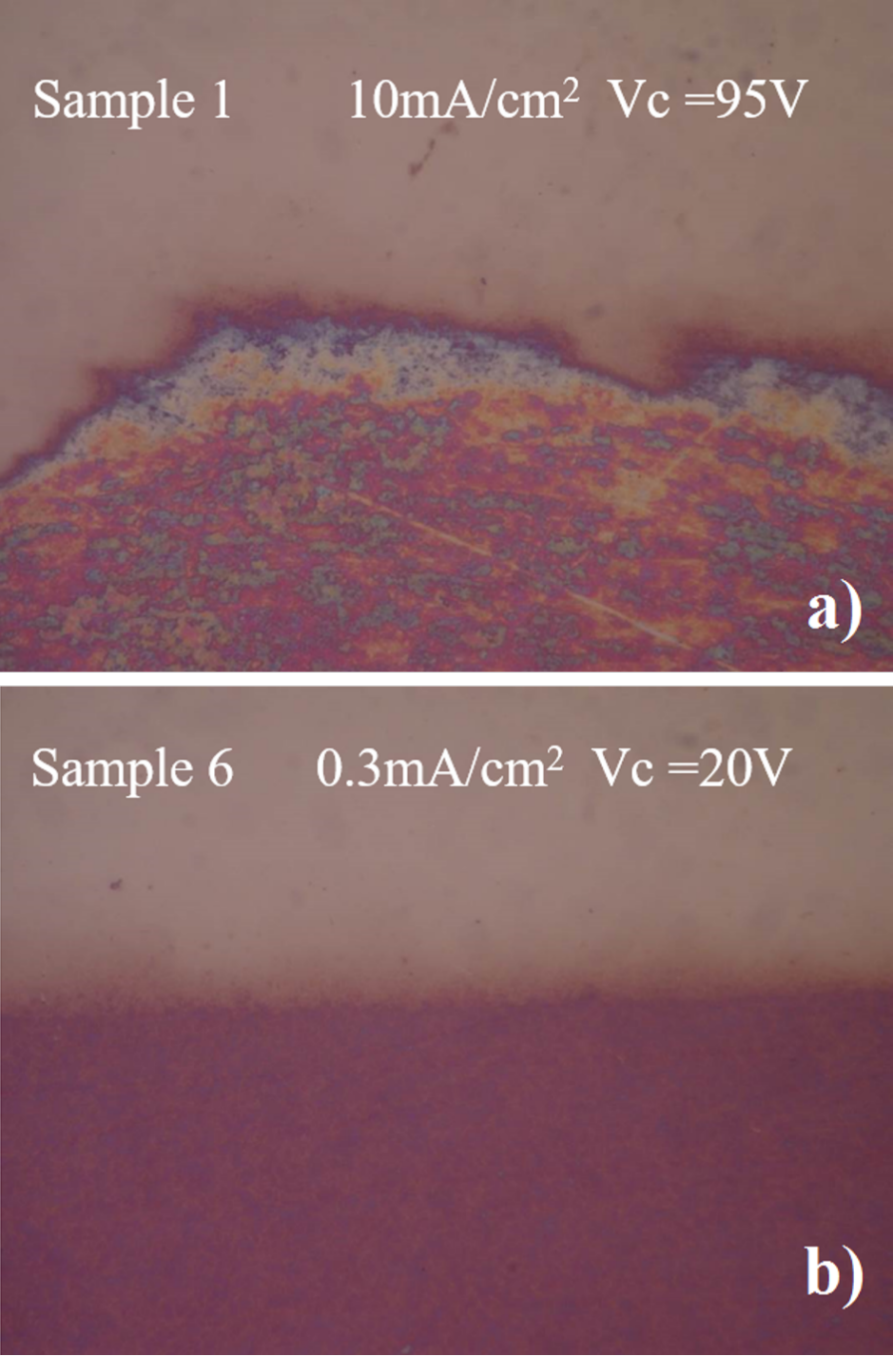
Optical microscopy images at 100× magnification of a) sample 1 in a) and b) sample 6. The upper part of the pictures is the NbN film, and the lower part is the oxided film after the anodization process.

Moreover, for these three samples (1,2,3) we clearly see changes in slope which is a signal of a non-uniform oxidation process, and of the fact that cracks and fractures are damaging the film. The existence of fractures and cracks is also confirmed from optical analysis of the surfaces. Indeed, after the oxidation the samples 1,2 and 3 the oxidized films were completely delaminated (Figure 8a). The visible contour separating the top from the bottom represents the separation between NbN (top) and the anodized Nb (bottom).

**Figure 9:**
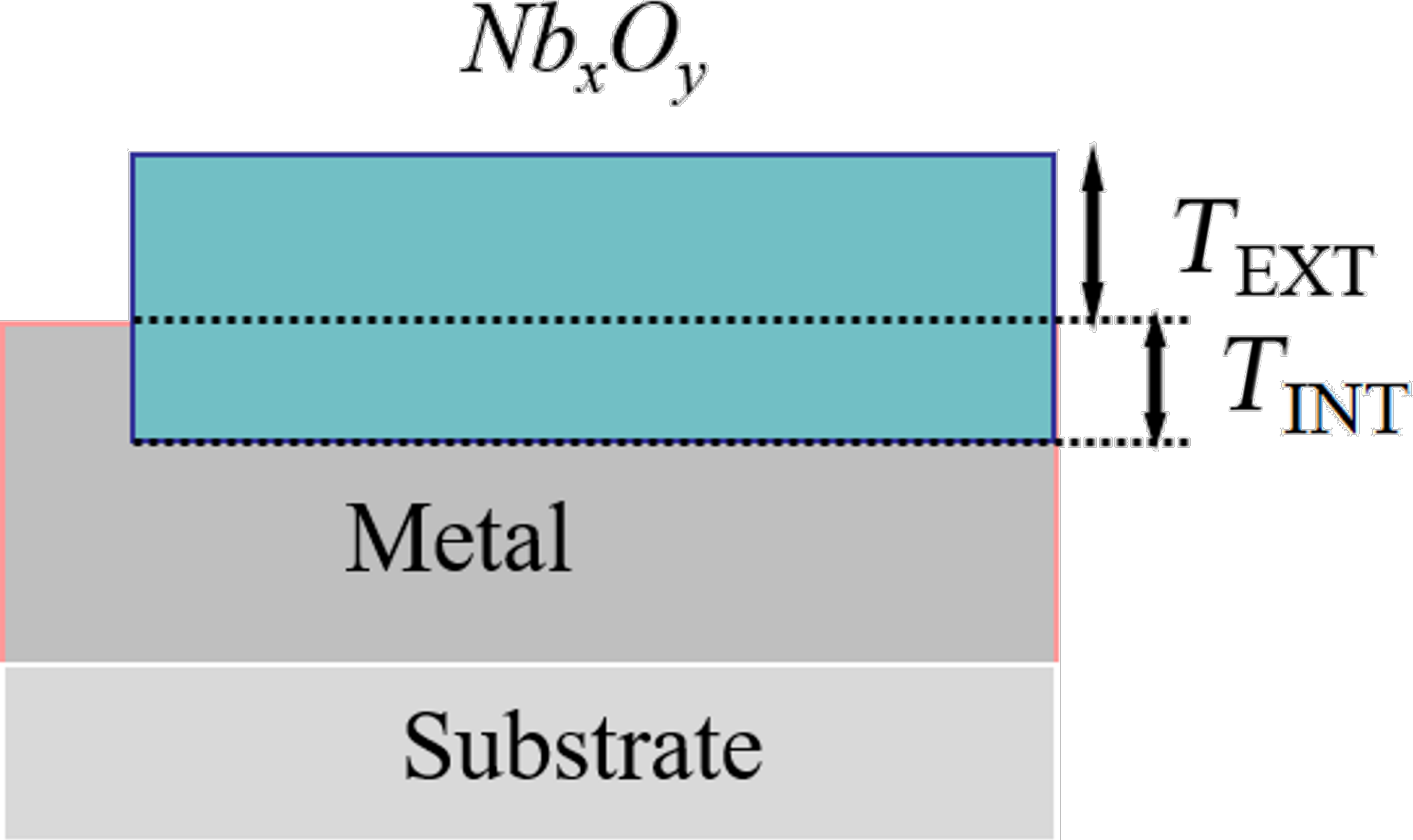
Scheme of the oxidized film grown on NbN. The total thickness of the oxide (*T*_EXT_ + *T*_INT_) is higher than the initial thickness of NbN (*T*_INT_).

Reducing the current density (curves 4 and 5) and *Vc* to 30 V, the fluctuations of *Vs* were reduced and the adhesion of the films was improved although the sample corresponding to curve 4 and 5 were still conducting. A careful analysis of these two curves in Figure 7 reveals that a change in slope is still present around 20–25 V. Further reducing *Vc* to 18–20 V we obtained curve 6, a smooth curve without voltage noise and change in slope. From an optical and electric analysis, the film is uniform, shows good adhesion and is an electric insulator (sheet resistance larger than 10 MΩ/sq). In Figure 8b we can see that the surface of the oxidized Nb (bottom part) is rather smooth and exhibits good adhesion (we shall later comment more on the mechanical properties). The oxide film grown on the NbN has a different final thickness due to different lattice parameters (Figure 9). NbN has a cubic structure (*Fm*−3*m*, space group 255, *a* = 0.44 nm, corresponding to a cell volume of 0.0846 nm^3^), whereas Nb*_x_*O*_y_* (the stable stoichiometry for an insulator should be Nb_2_O_5_) has an orthorhombic structure (*a* = 0.398 nm, *b* = 0.382 nm and *c* = 1.279 nm, cell volume of 0.194 nm^3^).

From the measurement of the step thickness and assuming a one-directional oxide growth (free space) we extracted the following relationship, where *T* denotes the thickness (see Figure 9 for the symbols): *T*_INT_/(*T*_EXT_ + *T*_INT_) ≈ 0.0846/0.194 and *T*_OX_ ≈ 1.8·*T*_EXT_.

It is possible to predict the thickness of the oxide layer measuring the height of the step. As a further relationship between the oxide thickness *T* and the voltage across the sample *Vs* we found *T* ≈ (2.8 nm)*V*_s_. This relationship could be the connection between the maximum voltage and the maximum thickness (ca. 60 nm) that we can grow before a crack or a fracture starts damaging the film. In the mechanical characterization, performed at 1 mN as maximum load, we observed continuous and smooth load-vs-depth graphs. The absence of cracks and delaminations during the measurements reveals good adhesion between the film and the substrate. When the composition of the layer changes from NbN to Nb*_x_*O*_y_* hardness and reduced modulus both decrease, respectively, from an average value of 5.9 GPa to 4.6 GPa and from 85 GPa to 78 GPa. The roughness of the oxidized surface was ±5 nm.

**Figure 10:**
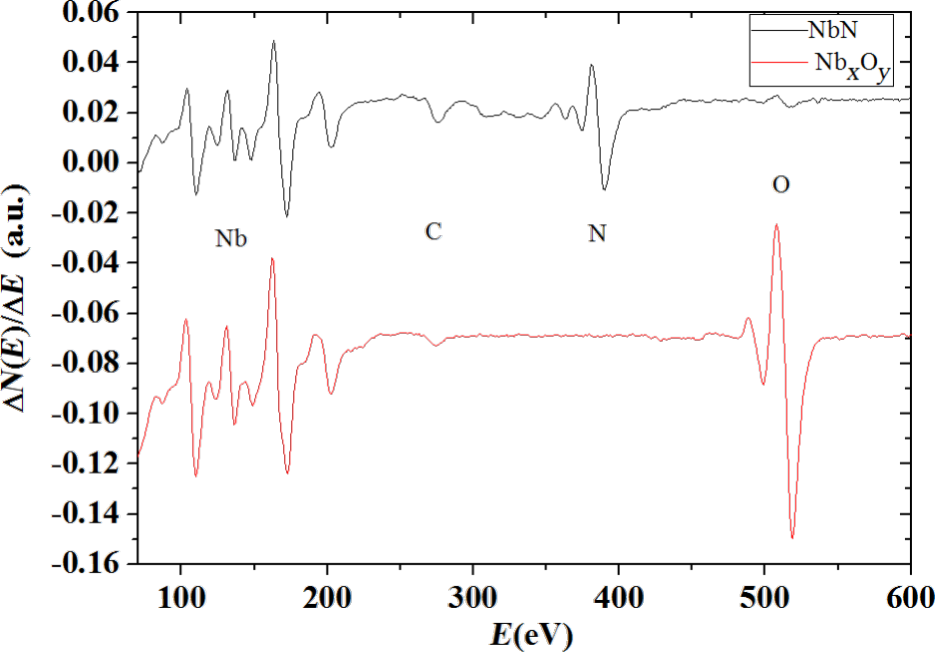
Auger electron spectroscopy of the initial NbN sample before and after anodization. A soft surface cleaning process by Ar ion etching was performed before the measurements. From the spectra is observed that only niobium and nitrogen are present before the process. After the oxidation process the nitrogen was completely replaced with oxygen.

To determine the chemical composition of the grown film we performed Auger electron spectroscopy (AES) and the spectra are shown in Figure 10. A complete nitrogen replacement with oxygen is observed from the measurements and a rough stoichiometry can be extrapolated compatible with Nb_2_O_5_.

### Tunnel junctions

**Figure 11:**
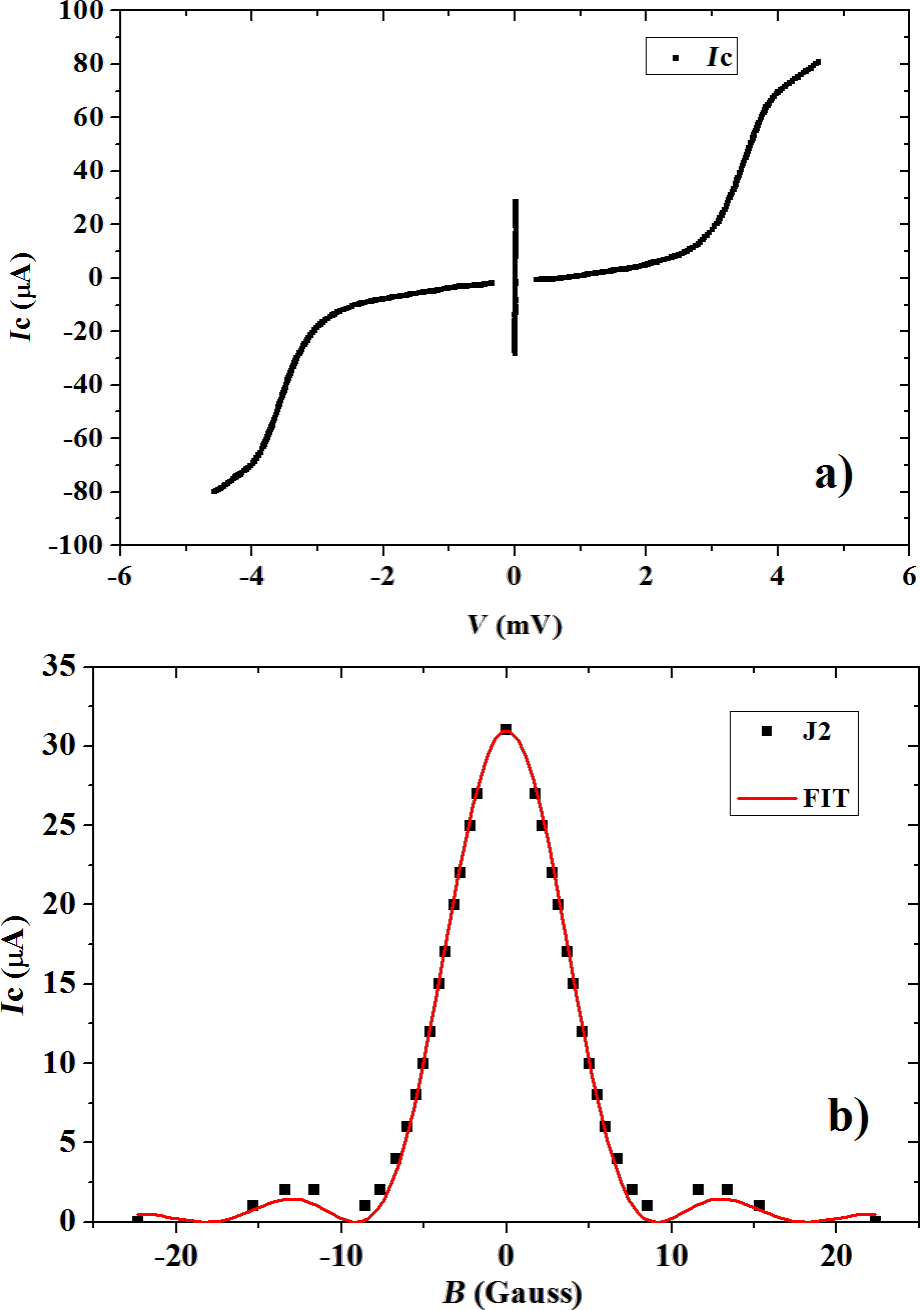
a) Current–voltage characteristic for NbN Josephson junction and b) diffraction pattern obtained measuring *J*c as function of the applied magnetic field. The continuous line is a fit obtained imposing a Josephson current distribution “rounded” along the corners of the nominally square area.

**Figure 12:**
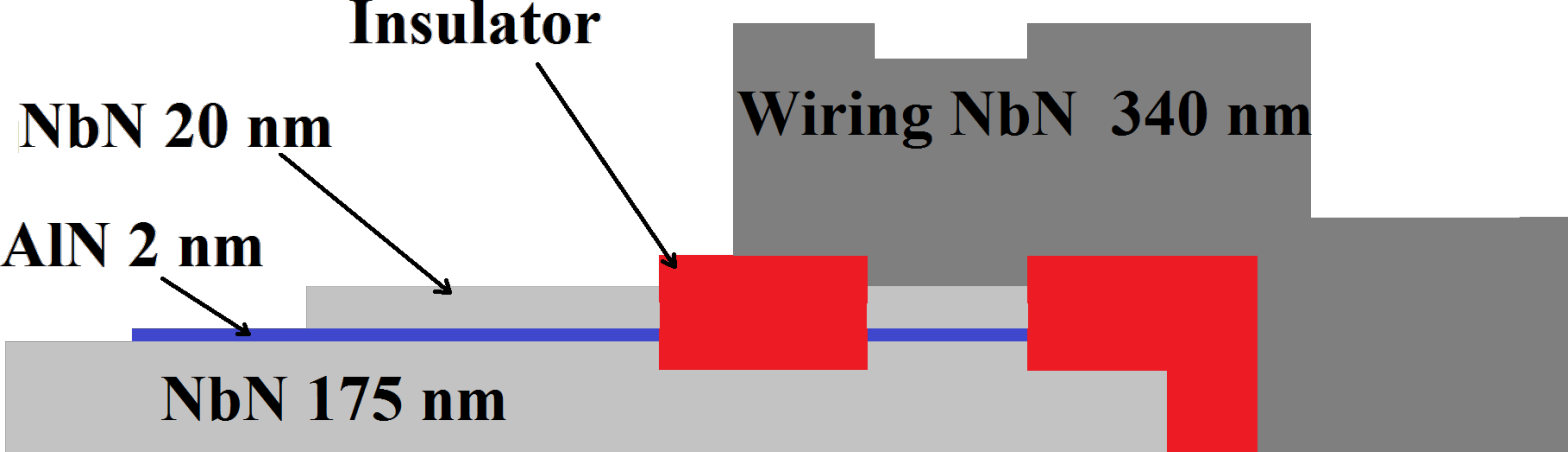
A section of our samples indicating the layers of the fabrication steps and their relative thicknesses.

In Figure 11a we show the current–voltage characteristic of a Josephson-junction measured at 4.2 K. The three-layer junction (NbN/AlN/NbN) is formed by three films of different thickness, namely 175 nm, 2 nm and 360 nm. The choice of the thicknesses of the films was imposed by design requirements. The Josephson junctions (Figure 12) were realized using UV lithography and a lift-off process. The (10 × 10) µm^2^ junction areas were defined anodizing the top layer following standard techniques [14]. The parameters of this junction are a representative result of our fabrication recipe: Josephson critical current density *j*_c_ = 14.2 A/cm^2^, product of maximum critical current and normal resistance *I*_c_*·R*_NN_ = 1.63 mV, *R*_NN_/*R*_SG_ = 10, *V*_m_ = *I*_C_*·R*_SG_ = 16.3 mV, gap sum voltage *V*_G_ = 3.6 mV. These parameters are sufficiently consistent with those reported by other research groups (10 A/cm^2^ < *j*_c_ < 8 kA/cm^2^, 1.7 mV < *I*_c_*·R*_NN_ < 3.32 mV, 5 < (*R*_NN_/*R*_SG_) < 40, 13 mV < *V*_m_ < 100 mV, gap sum voltage 4.2 mV < *V*_G_ < 5.6 mV [9–11]) thereby confirming the apparent impression of an overall reasonable result of our fabrication process. This conclusion is especially encouraging if we consider that the fabrication processes of other groups were carried out on crystalline substrates brought to high temperatures during deposition.

From the magnetic field diffraction pattern of Figure 11b we can extract the London penetration depth of our NbN films of 500 ± 30 nm. This result is in good agreement with previous results obtained through NbN deposition on non-heated substrates [18–19]. The pattern shows a somewhat non-uniform Josephson current distribution [20], which we speculate to be due to a reshaping of the contour of the junctions during the anodization process. The continuous line in Figure 11b is a fit to the data obtained by a non-uniform Josephson current distribution in the junction, namely a profile rounded around the corners of the supposed rectangular shape.

It is known that the growth of NbN/AlN/NbN tunnel junctions on oxidized silicon substrates can give rise to a non-optimal barrier formation. In order to check the influence of the substrate on trilayers we plan to test our trilayer process on MgO substrates, which are known, for providing the highest quality of the tunneling barrier [13]. We are not sure, however, that the nature of the substrate is the only crucial factor in fabricating NbN-based tunnel junctions with excellent quality. We have shown indeed that an acceptable (in terms of results) NbN process can be obtained just by lift-off lithography and anodization of the superconductive films grown over “cold” amorphous silicon oxide. Improvements of this recipe could substantially enhance the ease of fabrication and lead to noticeable progress in the scientific and technical usefulness of all-NbN tunneling devices.

## Conclusion

A viable method to optically monitor and tune, in a timely fashion, the chemical composition of the plasma in a sputtering process has been applied to grow thin films of niobium nitride and aluminium nitride. The advantage of this optical spectroscopy technique is that the control of the chemical composition of the film is no longer system-dependent and provides objective advantages during the reactive sputtering deposition. We have shown that for the NbN superconducting films, this method gives the possibility to increase or to reduce the flux of nitrogen and to obtain the best chemical composition within the constraints imposed by the other growth parameters. Similarly, it is possible to predict and obtain the conductive or insulating properties of AlN.

We have adapted the deposition process to the lithographic and technological needs for the realization of superconductor/insulator/superconductor and Josephson junctions devices. These techniques rely on three layers of NbN/AlN/NbN, and require, as a first step in the fabrication process the use of a photoresist-patterned substrate. This condition, in turn, limits the highest acceptable dc power to 200 W. For a reduced sputtering power, the plasma composition must be redefined, because only the right amount of N_2_ in the plasma composition gives the highest critical temperature of the NbN film. The highest transition temperature was reached for a ratio N_2_/Nb equal to 1.42 corresponding to a N_2_ flux of 9 sccm.

We have presented the required values for current density and compliance voltage to obtain a controlled and stable oxidation of a NbN thin film. Auger electron spectroscopy and nano-indentation analysis has been employed to verify respectively the complete oxidation of the surface and the mechanical stability of the film. We have also found a relationship between the growth of the oxide film and the voltage during the anodization as a method to control its thickness up to few angstroms per second. The properties and the quality of Josephson junctions obtained using our patterning based on lift-off lithography on cold substrates and the anodization of NbN for defining the junction area are not far from those reported by other groups for high-temperature deposition of the films and reactive etching for geometry definition.

## References

[1] Murduck J M (2001). Thin Films.

[2] Lucci M, Ren J, Sarwana S, Ottaviani I, Cirillo M, Badoni D, Salina G (2016). IEEE Trans Appl Supercond.

[3] Lucci M, Badoni D, Merlo V, Ottaviani I, Salina G, Cirillo M, Ustinov A V, Winkler D (2015). Phys Rev Lett.

[4] Nigro A, Nobile G, Palmieri V, Rubino G, Vaglio R (1988). Phys Scr.

[5] Dawson-Elli D F, Fung C A, Nordman J E (1991). IEEE Trans Magn.

[6] Noat Y, Cherkez V, Brun C, Cren T, Carbillet C, Debontridder F, Ilin K, Siegel M, Semenov A, Hübers H-W (2013). Phys Rev B.

[7] Chaudhuri S, Nevala M R, Maasilta I J (2013). Appl Phys Lett.

[8] Hazra D, Tsavdaris N, Jebari S, Grimm A, Blanchet F, Mercier F, Blanquet E, Chapelier C, Hofheinz M (2016). Supercond Sci Technol.

[9] Nagai Y, Akaike H, Kanada R, Naito N, Fujimaki A (2009). Supercond Sci Technol.

[10] Wang Z, Terai H, Kawakami A, Uzawa Y (1999). Supercond Sci Technol.

[11] Meckbach J M (2013). Superconducting Multilayer Technology for Josephson Devices.

[12] Gurvitch M, Washington M A, Huggins H A (1983). Appl Phys Lett.

[13] Lucci M, Sanna S, Contini G, Zema N, Merlo V, Salvato M, Thanh H N, Davoli I (2007). Surf Sci.

[14] Lucci M, Thanh H N, Davoli I (2008). Superlattices Microstruct.

[15] Zambrano G, Riascos H, Prieto P, Restrepo E, Devia A, Rincón C (2003). Surf Coat Technol.

[16] Henry F, Duluard C Y, Batan A, Reniers F (2012). Thin Solid Films.

[17] Pana I, Vitelaru C, Zoita N C, Braic M (2016). Plasma Processes Polym.

[18] Weihnacht M (1969). Phys Status Solidi.

[19] Kubo S, Asahi M, Hikita M, Igarashi M (1984). Appl Phys Lett.

[20] Barone A, Paternò G (1982). Physics and Applications of the Josephson Effect.

